# Floral temperature patterns can function as floral guides

**DOI:** 10.1007/s11829-020-09742-z

**Published:** 2020-01-13

**Authors:** Michael J. M. Harrap, Natalie Hempel de Ibarra, Heather M. Whitney, Sean A. Rands

**Affiliations:** 1 School of Biological Sciences, University of Bristol, 24 Tyndall Ave, Bristol, BS8 1TQ UK; 2 Centre for Research in Animal Behaviour, University of Exeter, Exeter, UK

**Keywords:** Pollination, Multimodality, Floral displays, Floral guides, Temperature patterns, Plant-pollinator evolution

## Abstract

Floral guides are signal patterns that lead pollinators to floral rewards after they have located the flower, and increase foraging efficiency and pollen transfer. Patterns of several floral signalling modalities, particularly colour patterns, have been identified as being able to function as floral guides. Floral temperature frequently shows patterns that can be used by bumblebees for locating and recognising the flower, but whether these temperature patterns can function as a floral guide has not been explored. Furthermore, how combined patterns (using multiple signalling modalities) affect floral guide function has only been investigated in a few modality combinations. We assessed how artificial flowers induce behaviours in bumblebees when rewards are indicated by unimodal temperature patterns, unimodal colour patterns or multimodal combinations of these. Bees visiting flowers with unimodal temperature patterns showed an increased probability of finding rewards and increased learning of reward location, compared to bees visiting flowers without patterns. However, flowers with contrasting unimodal colour patterns showed further guide-related behavioural changes in addition to these, such as reduced reward search times and attraction to the rewarding feeder without learning. This shows that temperature patterns alone can function as a floral guide, but with reduced efficiency. When temperature patterns were added to colour patterns, bees showed similar improvements in learning reward location and reducing their number of failed visits in addition to the responses seen to colour patterns. This demonstrates that temperature pattern guides can have beneficial effects on flower handling both when alone or alongside colour patterns.

**Electronic supplementary material:**

The online version of this article (10.1007/s11829-020-09742-z) contains supplementary material, which is available to authorized users.

## Introduction

Floral displays communicate with flower visitors through various signalling modalities at once. Such modalities include colour, scent, texture, temperature, and electrostatics in addition to patterns of these signals, where the composition or intensity of the signal differs across the floral display (Raguso 2004; Leonard et al. 2011, 2012). A possible explanation for this multimodality in floral displays is that additional signalling components convey different information to the pollinator: the ‘multiple messages hypothesis’ (Leonard et al. 2012). These floral messages could include information on flower identify, flower reward type or status (von Arx 2013). Additional signals might also provide spatial information about the flower. Certain floral signals may be used by pollinators to identify flower location in the environment, but others may indicate the location of rewards within the flower functioning as ‘floral guides’—sometimes known as ‘nectar guides’ (Sprengel 1793; Leonard and Papaj 2011; Hansen et al. 2012; Lawson et al. 2017a; Lawson and Rands 2018). Floral guides are contrasting signal patterns that help lead pollinators to the location of rewards within a flower. The most studied floral guides are colour patterns (Manning 1956; Daumer 1958; Dafni and Kevan 1996; Lunau et al. 2006; Hempel de Ibarra and Vorobyev 2009): these colour guides are found across many diverse floral taxa, and normally appear as radiating lines, speckles or solid blocks of contrasting colouring corresponding to the corolla entrance or nectary location. How other modalities function as non-visual floral guides is less well studied. Patterns of scent have been demonstrated to be capable of guiding bumblebees (Lawson et al. 2017a), and tactile patterns guide moth proboscis placement (Goyret and Raguso 2006; Goyret and Kelber 2011). Other signalling modalities show structured patterns, such as electrostatics (Clarke et al. 2013) and temperature (Harrap et al. 2017), but the capacity of these floral signalling modalities to function as floral guides has yet to be demonstrated.

Floral guides lead the pollinator to the rewarding region of the flower (Manning 1956; Daumer 1958; Goyret and Raguso 2006; Lunau et al. 2006; Leonard and Papaj 2011; Goyret and Kelber 2011; Hansen et al. 2012; Goodale et al. 2014; Lawson and Rands 2018). When compared to flowers lacking such patterns, floral guides have been reported to reduce the time spent searching for rewards on each flower (Leonard and Papaj 2011; Goyret and Kelber 2011; Goodale et al. 2014; Lawson et al. 2017a), increase incidence of flower visits where pollinators find floral rewards (Goyret and Raguso 2006; Goyret and Kelber 2011; Hansen et al. 2012), reduce the amount of time pollinators spend searching the flower after feeding (Leonard and Papaj 2011), and reduce the incidence of floral larceny (Leonard et al. 2013). Increasing the total amount of visits, the incidence of successful and legitimate (non-larceny) visits, as well as the speed at which visits take place, will all have beneficial consequences on pollen transport (Ushimaru et al. 2007; Benitez-Vieyra et al. 2007; Hansen et al. 2012). Furthermore, guides can help flowers control the direction of pollinator approach and its position while visiting, and this can allow plants to manoeuvre pollinators to a position that is best for pollen transfer (Ushimaru et al. 2007; Hansen et al. 2012).

Flowers often show radial gradients in temperature from the flower centre, near the nectary or on landing pad structures that protrude from corolla: 55% of 118 species sampled showed within-flower temperature differences that were greater than 2 °C (Harrap et al. 2017). Warmer flowers keep pollinator body temperature from dropping while they feed. In this way, floral temperature functions like a secondary floral reward by reducing the foraging costs of pollinators associated with maintaining body temperature (Rands and Whitney 2008). Consequently, many pollinating insects show a preference for elevated floral temperature (Seymour et al. 2003; Dyer et al. 2006; Sapir et al. 2006; Norgate et al. 2010). A preference for elevated temperature may attract pollinators to the warmer regions of flowers. Floral temperature patterns, where specific parts of the flower show elevated temperature compared to the rest of the flower, appear to be a common phenomenon (Rejšková et al. 2010; Dietrich and Körner 2014; Harrap et al. 2017, 2019). This combination of both distinct thermal patterning in flowers and pollinators showing both an ability to detect and a preference for warmer regions of a flower suggests that temperature patterns may be able to lead pollinators to floral rewards when the elevated temperature is localized to reward location. Temperature patterns may therefore function as a non-visual floral guide. Bumblebees are also able to learn floral temperature patterns (Harrap et al. 2017, 2019), so temperature patterns may allow further improved flower handling with experience.

Floral temperature is often the result of a flower’s ability to intercept solar radiation (Totland 1996), and will be strongly influenced by darker pigmentation and its patterning (Sapir et al. 2006; Rejšková et al. 2010; Harrap et al. 2017). Pollinating insect preferences for radial and darker floral colour patterns are well documented (Lehrer et al. 1995; Johnson and Dafni 1998; Hempel de Ibarra et al. 2001). These preferences are thought to cause the guided behaviours pollinators show at flowers with these patterns (Johnson and Dafni 1998; Goodale et al. 2014). However, temperature patterns can also occur without dark pigmented colour patterns, as floral temperature can be influenced by flower structure (Miller 1986), epidermal textures (Whitney et al. 2011), floral transpiration (Tsukaguchi et al. 2003), environmental temperature (Shrestha et al. 2018), or self-generated (thermogenic) heat (Seymour and Schultze-Motel 1997). Due to these associations between floral colour and temperature, pollinators visiting natural flowers with coloured guides are likely to encounter a range of temperature patterns that overlap in various ways with colour patterns—a multimodal display involving two modalities (temperature and colour, which are received by the pollinator using two different sensory modalities). Multimodal displays in other modalities are known to enhance floral learning beyond that of their unimodal components (Kulahci et al. 2008; Kaczorowski et al. 2012; Leonard and Masek 2014) or alter pollinator responses to other signals (Goyret et al. 2007, 2008), but is currently unknown whether a multimodal pattern that combines thermal and colour components functions better as a floral guide than colour alone.

To understand the guiding ability of natural flowers, we investigated whether floral temperature patterns function as a non-visual floral guide in a manner similar to tactile (Goyret and Raguso 2006; Goyret and Kelber 2011) and scent patterns (Lawson et al. 2017a). Using artificial flowers, we quantified several behaviours shown by visiting pollinators that have previously been shown to improve in response to floral guides (Goodale et al. 2014; Lawson et al. 2017a). Captive bumblebees were filmed over several visits to artificial flowers where reward location can be indicated by temperature, colour, or multimodal patterns. From this footage we analyse how our various flower-handling metrics differ between foragers on flowers with different patterns and those without such patterns. We predicted that the presence of temperature pattern would decrease search time over subsequent trials in both unimodal and multimodal flowers. If temperature patterns had an additive effect, then we expected to find further improvements over time and a strong prevalence of successful visits where a nectar reward is found.

## Methods

### Basic artificial flower construction

**Fig. 1:**
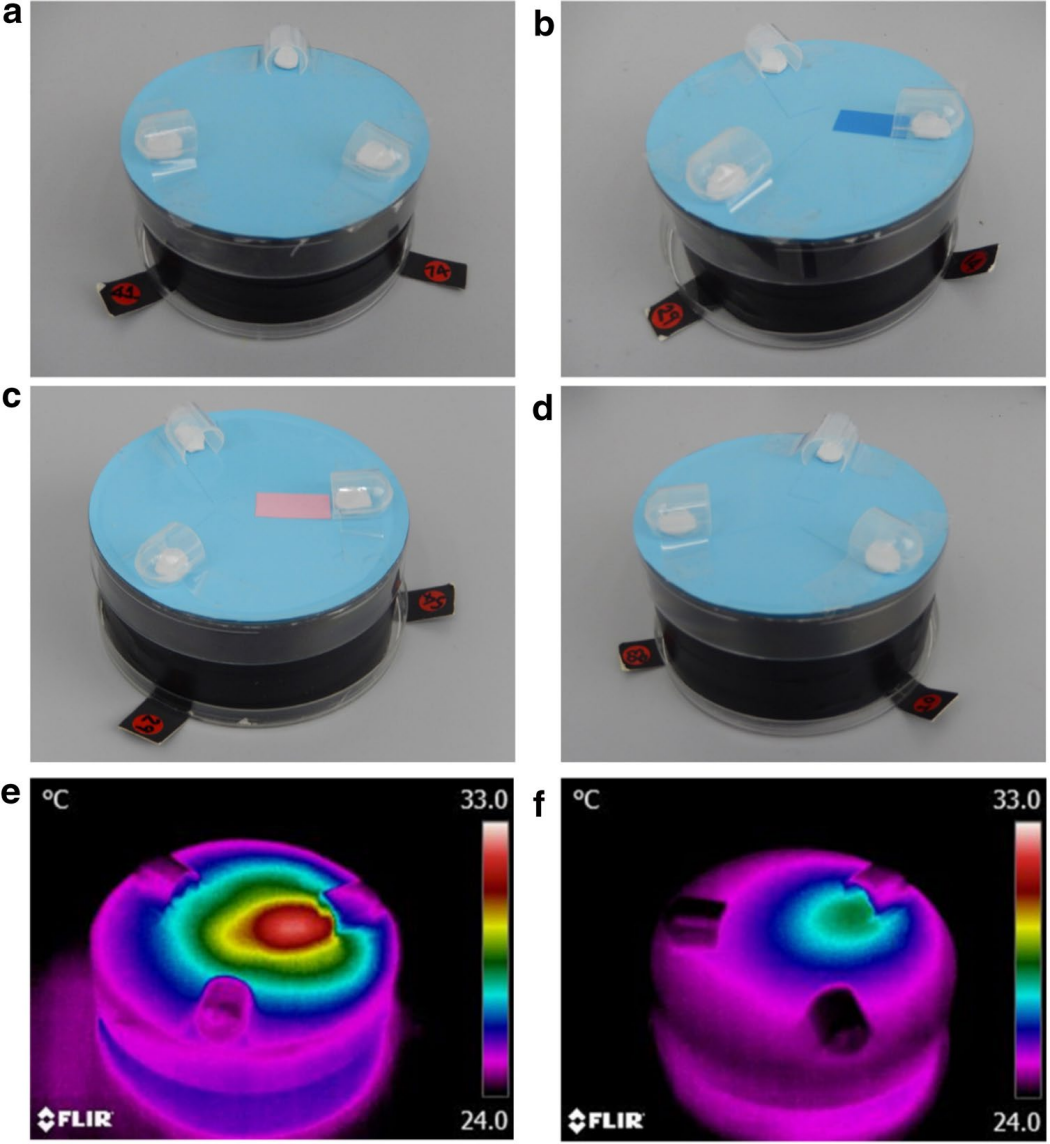
The colour and temperature patterns applied across artificial flower variants. **a** A test flower with no colour patterns and no control panels, as used in Plain Control, Unimodal Warm and Hot artificial flower variants. **b** A test flower with a blue colour panel, in front of the rewarding feeder, as used in test groups Unimodal and Multimodal Blue flower variants. **c** A test flower with a pink colour panel, in front of the rewarding feeder, as used in test groups Unimodal and Multimodal Pink flower variants. **d** A test flower with no colour pattern but with control panels, as used in the group ‘Panels Control’ flower variant. Note that the control panels are barely visible, but are present in front of all of the blue feeder tunnels in panels **b**–**d**. **e–f** Thermal images showing the heating that occurs when the ‘Hot’ (**c**) and ‘Warm’ (**f**) guide heating elements are turned on. The temperature scale in panels **e** and **f** is given in the colour scale to the right of each panel in °C. Note that both thermal images have the same temperature scale to allow comparison of the spread and amount of heating each thermal pattern generates

The artificial flowers were constructed from upturned petri dish bases (100 mm × 20 mm, *Sarstedt*, Nϋmbrecht, Germany) covered with aqua blue sticky back plastic (d-c-fix® adhesive film, Hornschuch group, Weissbach, Germany). The thermal emissivity of the plastic was measured, using electrical tape as a standard emissivity reference. The emissivity of the plastic was 0.95 (the same emissivity as the electrical tape), and this value was used for all measurements conducted, following the recommendations given by Harrap et al. (2018). Feeding wells were made from upturned Eppendorf tube lids (Multiply-pro cup 0.2 mlPP, Sarstedt, Germany) stuck on a sheet of thermally insulating 1 mm thick white plastic foam. Three feeding wells were stuck on each flower at 5 mm from the edge, orientated so that the lids’ cap pointed outwards at 120° angles from each other (Fig. 1a).

Each flower top was supported on a 42 mm tall, 85 mm diameter card cylinder wrapped in black electrical tape. Three 3 × 1 cm card rectangles were stuck at 120° angles to the top of the lid of the petri dish, which was used as the underside of the bottom of the artificial flower (Fig. 1), such that 2 cm tabs extended from the dish’s edge. A circular red sticker with a two-digit number written in black ink was stuck to each of the card tabs. Numbering allowed the researcher to identify rewarding and unrewarding feeders without giving meaningful cues to the bees, and followed the protocol described by Harrap et al. (2017).

The bulbs of 3 ml plastic pipettes (Pastettes, Alphalaboratories, UK) were cut down to create a 16 mm plastic hood-shaped tunnel, as used by Pearce et al. (2017) and Lawson et al. (2017a). These bulbs were placed over the feeding wells so that the open end of the tunnel faced into the flower centre (Fig. 1), limiting the bees to only approach the well from the centre of the flower. These were taped down at the beginning of testing each day with a fresh section of clear tape (*Scotch Easy Tear*, St. Pauls, USA).

### Artificial flower patterns

Colour or temperature patterns were added to the basic construction of the experimental artificial flowers described above. Two types of temperature patterns and two types of visual patterns were used in this experiment.

Colour patterns were created by sticking down a 1 × 2 cm adhesive d-c-fix® plastic panel in front of feeder tunnels. Two types of contrasting visual guide panels were used: ‘Blue panels’, which were a darker shade of blue than the flower top (Fig. 1b), and pale pink ‘Pink panels’ (Fig. 1c). To control for tactile effects caused by these coloured portions being stuck on the flower and therefore being slightly raised with a possibly tactile edge, we added ‘Control panels’ in front of feeder tunnels that did not have a blue or pink panel stuck in front of them. These control panels were made of the same aqua blue plastic as the flower, and therefore presented a similar tactile effect to the coloured panels (controlling for the bees being able to feel the raised edges of the plastic when standing on the flower), but without any contrasting colour to the background. An additional control was also created where all three tunnels had the control panel in front of them (Fig. 1d). The reflectance spectra of the coloured panels are described in Appendix S1 (Supplementary Material).

Temperature patterns corresponding with reward location were generated using heating elements placed on the underside of the flower, following a similar design to Harrap et al. (2017, 2019). Two kinds of temperature patterns were used, identified here as ‘Hot’ or ‘Warm’ (Fig. 1e–f). Construction is described in Appendix S2 (Supplementary Material). These two patterns differed in the level of temperature generated, however both patterns show within-flower temperature contrasts comparable to natural flowers (Harrap et al. 2017). In ‘Hot’ artificial flowers, surface temperature settled at approximately 30 °C in front of the ‘Hot’ feeder and 26 °C in front of the cooler ones, giving a temperature difference of 5–6 °C across the flower. In ‘Warm’ artificial flowers, surface temperature settled at approximately 28 °C in front of the ‘Warm’ feeder and 24 °C in front of the cooler ones, giving a temperature difference of 4–5 °C across the flower.

Using combinations of the guides described above, eight different variants of experimental artificial flowers were constructed, as described in Table 1. These included two controls, two unimodal colour guides, two unimodal temperature guides, and two multimodal patterns with both a temperature and colour guide present.

**Table 1:** The artificial flower variants, and additionally the experimental test groups, used in experiments

Artificial flower	Number of bees	Floral patterns	
Plain control	12	Rewarding feeder:	No colour or heating
		Nonrewarding feeders:	No colour or heating
Panels control	12	Rewarding feeder:	Control panel; No heating
		Nonrewarding feeders:	Control panel; No heating
Unimodal pink	12	Rewarding feeder:	Pink panel; No heating
		Nonrewarding feeders:	Control panel; No heating
Unimodal blue	12	Rewarding feeder:	Blue panel; No heating
		Nonrewarding feeders:	Control panel; No heating
Unimodal warm	12	Rewarding feeder:	Warm thermal pattern; No colour
		Nonrewarding feeders:	No colour or heating
Unimodal hot	12	Rewarding feeder:	Hot thermal pattern; No colour
		Nonrewarding feeders:	No colour or heating
Multimodal pink	12	Rewarding feeder:	Pink panel; Warm thermal pattern
		Nonrewarding feeders:	Control panel; No heating
Multimodal blue	12	Rewarding feeder:	Blue panel; Warm thermal pattern
		Nonrewarding feeders:	Control panel; No thermal heating

Each flower type is listed and the signals associated with rewarding and nonrewarding feeders are given. Additionally, the number of bees presented with each artificial flower variant during the test phase, alternatively the number in each test group, are given

### Pre-training period

Bee lab experimental techniques were used to investigate bumblebee guide responses and handling of artificial flowers. Flower naïve bumblebees, *Bombus terrestris audax*, were supplied by Biobest (Westerlo, Belgium) via Agralan (Swindon, UK). General husbandry, marking procedures and flight arena design is described by Lawson et al (2017a, b).

Outside of the testing period, bees were fed sucrose solution daily from PCR racks, gravity feeders and a selection of ‘generic’ artificial flowers placed within their flight arena, to ensure that bees learnt to handle feeding wells on arbitrary artificial flowers. Most generic artificial flowers were constructed from a 44 mm wide specimen jar (Sterilin PS 60 ml, with white plastic lids, Thermo Fisher Scientific, Newport UK), or resin disks of a similar size, with single feeding wells stuck to them. At least a week prior to bee trials, some of these generic artificial flowers were substituted with flowers to prepare bees for this experiment. These flowers showed one or two of the following: a different size (being either made from a larger specimen jar or a petri dish); multiple feeders; feeders not in the centre of the flower; or tunnels over the feeder (constructed as described above). These ‘new’ generic artificial flowers allowed bees to get used to feeding from flowers showing aspects of those used in this experiment. However, none of these new generic artificial flowers showed all these aspects together. Furthermore, these new generic artificial flowers never showed feeders at fixed angles about the flower edge, and never showed any visual or temperature patterns, or any colours associated with visual patterns. Additionally, other generic artificial flowers were still present and made up the majority (four out of seven or eight) of artificial flowers presented outside of trials.

Preliminary trials found that the experimental artificial flowers were too complicated for bees to learn to use in a single visit. Naïve bumblebees did not land on the experimental flowers described above, and so a pre-training phase with simplified flowers ‘pre-training artificial flowers’ was therefore included before the training period and test itself. A pre-training phase allowed bees to learn how to feed from artificial flowers similar to experimental flowers without gaining direct experience on the experimental flower or patterned signals. The top of these pre-training flowers was a petri dish lid covered with the same aqua blue sticky back plastic as the test flowers. Three feeding wells with foam bottoms were stuck to the top of the artificial flower, as in the test flowers (described above), but the sides of the Eppendorf tube lids were painted black. This lid was then supported on a 55 mm tall card cylinder, wrapped in black electrical tape and taped to the outside of the petri dish lid. Pre-training flowers did not possess tunnels, visual or temperature patterns, a base, or feeder labels.

During pre-training, marked forager bees were released into a foraging arena containing a clean pair of pre-training flowers with a droplet of 30% sucrose solutions in all their feeders. These pre-training flowers were placed in the centre of the foraging arena about 30 cm apart from each other in line with the bee’s entrance to the arena. A camcorder (Legria HF r36; *Canon*, Tokyo, Japan) was placed above each of the pre-training flowers. Each camcorder had a wide-angle lens attachment (XIT pro series 0.43X HD wide-angle lens 52 mm, *Xit Group*, Brooklyn, USA), placed facing down to view the artificial flower. Though pre-training was not recorded, the camcorder was present in pre-training so bees acclimated to it.

Multiple bees could be released into the flight arena together during pre-training. Bees were allowed to feed freely and return to the nest at will, with feeders being refilled when empty. If a bee completed two foraging bouts feeding on the pre-training flowers (departing and returning to the nest being one bout), it was deemed to have completed the pre-training phase. On a given sampling day, bees that had completed pre-training that day could then be used in the test phase. Most bees (69%) that began the pre-training phase went on to complete it. The bees that failed pre-training will include forgers unable to manipulate these artificial flowers, but also erroneously marked non-forager bees. If a bee completed pre-training but was not used for the test phase that day (for example because other bees took too long to complete testing), it could be used another day but would need to recomplete the pre-training phase. If a bee began the test phase it had to complete it in a single day. Bees that began the test phase were never reused in this experiment, even if they did not complete the test phase.

### Test phase

Following completion of the pre-training phase, bees were assigned artificial flower variants as described in Table 1. Individual bees were presented one variant of experimental artificial flower throughout the test phase. Consequently, the variant of artificial flower that a bee was presented with also describes its experimental test group. Throughout the experiment, temperature patterns were monitored by a FLIR e60bx thermal camera (FLIR systems, Inc., Wilsonville, USA). Before the bee began a foraging bout, the rewarding and unrewarding feeders (described in Table 1) of 8 experimental flowers were filled with 25 µl of 30% (volume-to-volume) sucrose solution or water respectively, using an electronic pipette (HandyStep® Electronic, Wartheim, Germany).

During the test phase, bees that had completed the pre-training phase that day were allowed to make successive foraging bouts on their assigned test flowers. Each test bee foraged alone in the arena during the test phase, and other bees would not be released into the arena during testing. When a bee began a foraging bout, a single artificial flower of the variant assigned to that bee was present in the arena. On the first foraging bout, this first artificial flower was placed in the same position as one of the pre-training flowers had been. The bee was allowed to land and forage on the artificial flower. Once a bee had extended its proboscis into any of the test flower’s feeding wells (recorded as a ‘drinking event’), a fresh artificial flower was placed in the arena at least 15 cm away from the bee. If the bee had drunk from any of the feeding wells on a flower, that flower was removed from the arena after the bee had departed. Bees were acclimated to the removal and insertion of artificial flowers into the arena, as this was done outside to testing for feeding. Thus, it is unlikely they were disturbed by flower removal and placement.

A ‘visit’ began when bees first made physical contact with artificial flowers. As artificial flowers were quite large, bees often flew from one part of the flower to another when searching. Thus, classing departure from the flower simply as the moment a bee broke contact with a flower after landing would not be representative of the bee’s searching effort and would often result in many aborted landings occurring before the first feeding. For this reason, a bee was classed as ‘departing’ if it broke contact with any part of the flower, then either flew away from the flower and did not return within 5 s, or flew over 30 cm away from the flower, or landed on another. These criteria allowed for bees to fly from one part of the flower or hover about the flower after landing without being classed as departing when they were still apparently searching the flower. Additionally, these criteria allowed for bees to climb about and search the lower parts and sides of the flower without being classed as departing. A ‘visit’ was assumed to end when the bee met one of these departure criteria.

Upon the bee’s departure, the flower that had just been drunk from was immediately removed in order to avoid the bee becoming satiated or distracted. This exchange of flowers (placing a new one inside the arena once the bee fed and removal upon the bee’s departure) continued until the bee returned to the nestbox on its own accord or had fed from all eight experimental artificial flowers in a single foraging bout. Once a bee had departed from the eighth flower in a bout, the eighth flower was removed and no more flowers were placed in the arena in that bout. The bee then returned (or was returned) to the nestbox.

Artificial flowers were reused in subsequent foraging bouts. Once a bee had completed a foraging bout, all flowers were removed from the flight arena. Any water and sucrose solution left in the feeding wells of flowers visited in the previous bout were emptied using paper towel. Flowers were then wiped down with ethanol, removing scent marks that may conflate bee decisions (Stout and Goulson 2001; Pearce et al. 2017). The feeding wells of these flowers were then refilled. The cycle of removal and replenishing of water and sucrose between bouts reduced the chance of differences in the temperature of the feeding well contents developing. Flower temperature was checked with the thermal camera and any flower that had overheated or ceased to produce a temperature pattern was replaced. After cleaning, thermal signals were allowed to re-settle before re-use, as ethanol evaporation cools flowers.

Before the bee was let back into the arena an artificial flower was placed inside. Bee foraging was then allowed to continue as described above. The first artificial flower placed in the arena in bouts after the initial foraging bout were placed anywhere in the arena, rather than the same positions of pre-training flowers. Individual bees were allowed to carry out successive bouts of foraging until the bee completed the bout where the number of flowers visited across all bouts was at least 30. At this point the bee was deemed to have completed the test phase.

Occasionally bees were reluctant to visit the flower in the arena. To encourage the bee, another artificial flower would be placed into the arena. In this scenario, if a bee drank from and departed from either flower, that flower would be removed but a new flower would not be placed inside the arena, as one was already present. Otherwise the experiment carried on as normal. If there were already two artificial flowers within the arena and bees still seemed reluctant to visit an artificial flower, an artificial flower would be moved to a new position. Moving flowers would not be carried out if the bee had already visited a flower but not fed. At any one time there were never more than two artificial flowers in the arena.

Video cameras were used to record bee flower visits and flower handling in the test phase. Whenever an artificial flower was placed in the arena, either at the start or during a foraging bout, a camera that was not already viewing a flower would be moved into position above it. This was done immediately after a flower was placed in the arena. Viewing the artificial flowers from above meant that the entire flower top and at least two of the numbered tags at the bottom of the flower were visible.

96 bees from 14 nestboxes completed the test phase, with 12 in each of the 8 test groups (Table 1). Bees completed the test phase in 31.59 ± 0.01 visits (mean ± SEM). However, due to camera recording error the visits after focal visit 29 for bee 5 (in Plain Control group) and 84 (in Multimodal Pink group) were not recorded, similarly visit 30 (of 33) of bee 81 (in Multimodal Pink group) was not recorded due to an error in camera placement. This meant the number of filmed visits for each bee ranged from 29 to 35. The test phases of all bees were carried out between 10:30 and 18:30, the normal time period of bumblebee foraging activity.

### Data processing

Data on flower handling was collected by reviewing the recordings of a bee’s behaviour during visits to experimental artificial flowers. Flower handling data was only collected with reference to a ‘focal flower visit’, defined as a flower visit during which a bee first drinks from any feeding well on the flower, or the last visit a bee makes on a flower where feeding wells are never drank from. This means that we ignored visits prior to the focal visit where the bee does not feed, and also ignored return visits to the flower after the focal visit (in instances where the flower could not be removed in time).

Three metrics were collected for each bee. All metrics were collected by a single observer (MJMH) for internal consistency.

1. *Proportion of failed visits* Floral guides have previously been shown to reduce the proportion of failed visits made by the pollinator (Goyret et al. 2007; Hansen et al. 2012). For each bee, we calculated this as the proportion of focal visits where the bee failed to find rewards (‘failed visits’) in the previous 10 focal flower visits at 10, 20 and 30 focal visits (so calculating the proportions for visits 1–10, 11–20 and 21–30). In instances were camera errors meant focal visit 30 was not recorded, noted above, the proportions of failed visits at 30 focal visits for these bees were calculated using the previous 9 visits recorded, but were otherwise treated the same. For these proportions, focal visits made after visit 30 were ignored for analyses.
2. *Proportion of first-feeder visits* Floral guides have been identified to indicate reward locations and draw pollinators to them, which can lead to an increased incidence of visits to a rewarding feeder immediately upon sampling a flower (Johnson and Dafni 1998; Lunau et al. 2006; Goodale et al. 2014). We measured these in an identical manner to the proportion of failed visits, except considering the proportion of focal visits where the rewarding feeder was the first on a flower that a visiting bee chose to drink from.
3. *Reward search time* Floral guides have previously been shown to reduce the length of reward search time (Leonard and Papaj 2011; Goyret and Kelber 2011; Lawson et al. 2017a). During each focal visit, it was recorded whether bees found the rewarding feeders on the first, second, or third feeder they drank from (ignoring revisits to drink again from the same feeder), or whether they departed after having failed to feed on the rewarding feeder. On focal flower visits where rewards were successfully found (a ‘successful visit’), the reward search time was recorded as the time between the start of the focal visit and the start of the first drinking event from the rewarding feeder. This was measured with a stopwatch while replaying video in real time, as done in Lawson et al. (2017a). All data on reward search times was used in analyses, including recorded focal visits that occurred after 30 visits.

### Statistical analysis

All data were analysed using *R* 3.4.1 (R Development Core Team 2017). Proportions of failed visits and first-feeder visits were arcsine square root transformed. How proportions of failed visits, proportions of first-feeder visits and reward search time were altered by the floral patterns presented to each bee, and how these changed with experience (the number of focal visits) were analysed independently using a generalised linear models and AIC model simplification techniques. This involves a sequential process of paired comparisons between a standing ‘best model’ and a simpler model fitted to the data using AIC. Simpler models were constructed by removal of parameters from the standing best model (forcing parameters to equal zero). If removal of parameters resulted in a significant increase in AIC, based on Richards (2008), the standing best (more complex) model would remain the best for the next comparison. If otherwise, simpler models would become the standing best model for the next comparison. The best fitting model being the one remaining at the end of the sequence of comparisons. Each metric was assessed independently, through the same sequence as described in detail in Appendix S3 (Supplementary Material).

## Results

### Proportion of failed visits

**Fig. 2:**
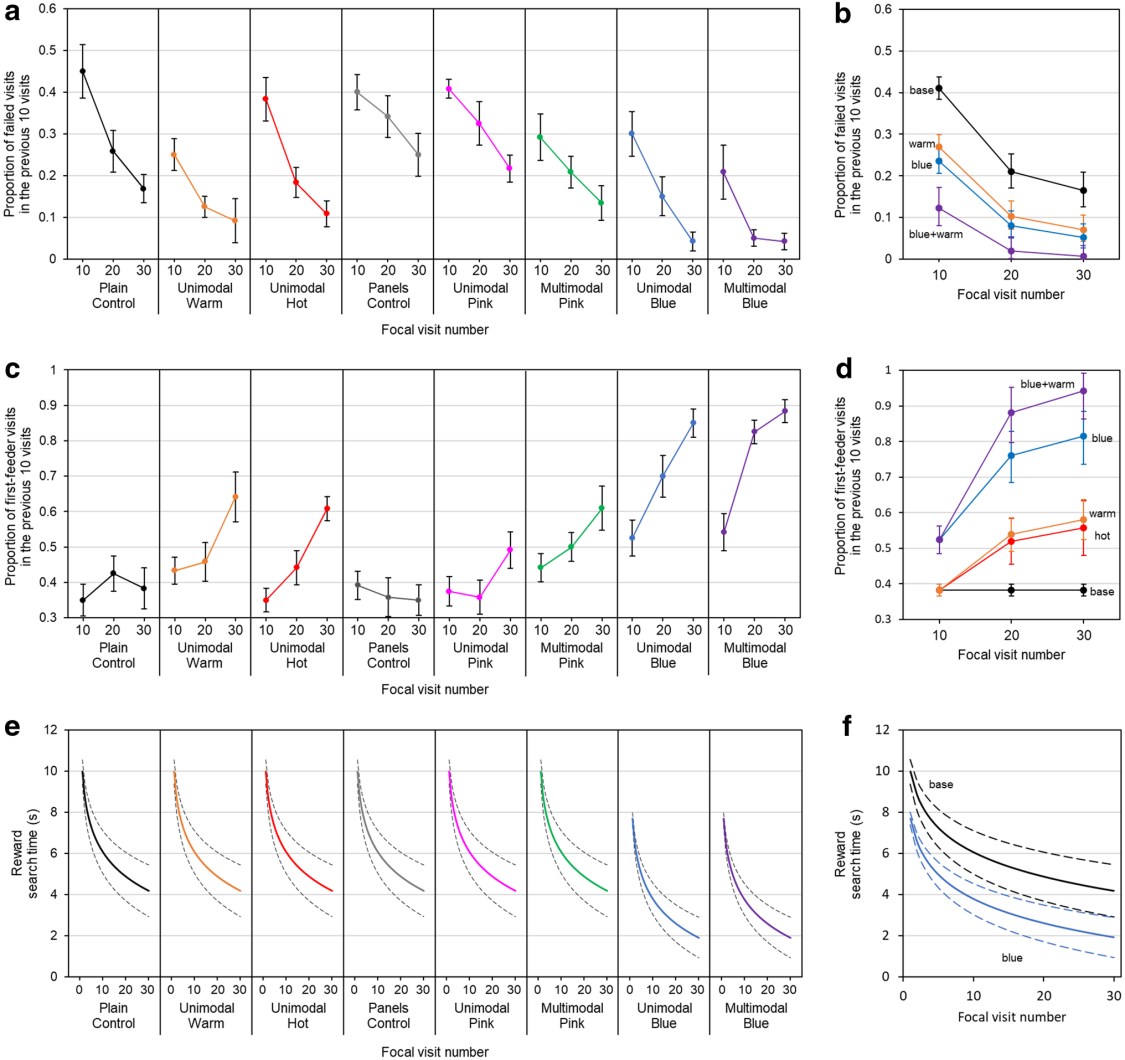
The relationships between each handling metric and experience (focal visit number) across bees that foraged on different artificial flower variants, different test groups. **a** The mean proportions of failed visits (proportions are calculated for the previous ten focal visits at ten, twenty and thirty focal flower visits) for bees in each test group. Error bars indicate ± one standard error of the mean. **b** A summary of the best fitting model for proportions of failed visits for bees foraging across all flower test groups. Points plotted are the mean proportions of failed visits for bees presented with different pattern or pattern combinations, as predicted by the best fitting model. Error bars indicate ± one standard error of the model mean estimates. **c** The mean proportion of first-feeder visits for bees in each test group. Error bars indicate ± one standard error of the mean. **d** A summary of the best fitting model for proportions of first-feeder visits for bees foraging across all flower test groups. Points plotted are the mean proportions of first-feeder visits for bees presented with different pattern or pattern combinations, as predicted by the best fitting model. Error bars indicate ± one standard error of the model mean estimates. **e** Solid lines indicate the mean reward search times for bees in each test group as predicted by the best fitting search time model. Dashed lines indicate ± one standard error of the model mean estimates.** f** A summary of the best fitting model for reward search times of bees foraging across all flower test groups. Solid lines indicate the mean reward search times for bees presented with different patterns or combinations of patterns, as predicted by the best fitting model. Dashed lines indicate ± one standard error of the model mean estimates. Across panels **b**, **d** and **f**, colours and labels indicate the patterns presented to bees: ‘blue + warm’ and purple colour, bees presented with both Warm temperature and Blue colour patterns together; ‘blue’ and blue colour, indicates bees presented with blue colour patterns; ‘warm’ and orange colour, bees presented with warm temperature patterns; ‘hot’ and red colour, bees presented with Hot temperature patterns; ‘base’ and black colour, bees not presented with patterns (control groups) or patterns not indicated by any other line, thus ‘base’ includes bees whose responses were indistinguishable from control bees

Proportions of failed visits by bees across test groups are shown in Fig. 2a. Comparisons of the control groups found both groups showed similar relationships with experience and similar initial proportions of failed visits (*Interaction term*: standing best model AIC − 5.91, simpler model AIC − 6.70, ΔAIC = 0.78, Δdeviance = 1.21, df = 1, *p* = 0.270, *Intercepts*: standing best model AIC − 6.70, simpler model AIC − 8.16, ΔAIC = 1.46, Δdeviance = 0.53, df = 1, *p* = 0.464).

The results of the model selection process of proportions of failed visits across all test groups are summarised in Table S1 (Supplementary Material). Bees showed consistent changes in proportions of failed visits with experience (Fig. 2a, b) regardless of the patterns presented to them. Bees presented with Blue or Warm temperature patterns had lower intercepts compared to other bees. Hot or Pink patterns had no significant effect on visit failure. No interaction effects between Blue and Warm patterns, or Pink and Warm patterns were found. This meant that the best fitting model favoured a further reduction in proportion of failed visits for bees presented with both Warm and Blue patterns compared to bees presented those patterns alone (Fig. 2b).

### Proportion of first-first feeder visits

Proportions of first-feeder visits of bees across test groups are given in Fig. 2c. Comparisons of the control groups found both groups showed similar relationships with experience and similar overall proportions of first-feeder visits (*Interaction term*: separate group model AIC − 13.29 *vs.* equivalent group AIC − 14.23, ΔAIC = 0.94, Δdeviance = 1.06, df = 1, *p* = 0.304, *Intercepts*: separate group model AIC − 14.23 *vs.* equivalent group AIC − 16.18, ΔAIC = 1.95, Δdeviance = 0.05, df = 1, *p* = 0.827).

The results of the model selection process for proportions of first-feeder visits across test groups are summarised in Table S2 (Supplementary Material). The best fitting model found bees presented with Blue patterns showed a higher intercept and increased proportions of first-feeder visits with experience. Bees presented with Hot or Warm temperature patterns increased in proportions of first-feeder visits with experience but showed no altered intercepts (Fig. 2c–d). Pink patterns had no effect on bees. Bees not presented with Blue, Hot or Warm patterns appeared to maintain a constant proportion of first-feeder visits, comparable with random choice of which feeder to feed from first. No interaction effects between Blue and Warm patterns, or Pink and Warm patterns were found. As Warm patterns had no influence on intercepts this meant bees presented with Blue and Warm patterns together had similar initial probabilities of first-feeder visits (model intercept) to bees presented with Blue patterns alone, but with experience bees presented with multimodal patterns showed a greater incidence of first-feeder visits.

### Reward search time

Reward search times across test groups are summarised in Fig. 2e. Comparisons of the control groups found both groups showed similar relationships with experience and similar overall first-feeder rates (*Interaction term*: standing best model AIC 3431.5 simpler model AIC 3432.2, ΔAIC = 0.7, Δdeviance = 2.72, df = 1, *p* = 0.099. *Intercepts*: standing best model AIC 3432.2 simpler model AIC AIC 3430.5, ΔAIC = 1.7, Δdeviance = 0.30, df = 1, *p* = 0.585).

The model selection process is summarised in Table S3 (Supplementary Material). The best fitting model found bees presented with Blue patterns showed reduced reward search times (Fig. 2e–f). No other effects of any other patterns or interactions between patterns (multimodal effects) on reward search time were found.

## Discussion

Our study shows that temperature patterns can induce some changes in bumblebee behaviour (Fig. 2). Bees visiting flowers presenting Warm temperature patterns showed reduced incidence of visits where they failed to find rewards (failed visits, Fig. 2a, b) and improved learning of the floral reward location (Fig. 2c, d), as shown by increases in the proportion of visits where they fed from the rewarding feeder first (first-feeder visits) with experience. Hot temperature patterns allowed similar learning of reward locations, but did not influence the incidence of failed visits. When presented alone, pink colour patterns appeared to have no influence on bee foraging responses (Fig. 2). This is likely to be due to pink colour patterns showing little contrast with the flower top and are not easily detected by bees (Supplementary material, Appendix S1). Contrasting colour patterns, blue colour guides, induced changes in bee behaviour in all the handling metrics assessed, as expected for a contrasting colour guide (Lunau et al. 2006; Leonard and Papaj 2011; Hansen et al. 2012; Goodale et al. 2014). Bees visiting flowers with blue colour patterns had reduced search time for floral rewards (Fig. 2e, f) and reduced proportions of failed visits (Fig. 2a, b). Additionally, bees presented with blue patterns showed increased initial proportions of first-feeder visits (Fig. 2c, d) indicating bees were showing an innate tendency to investigate the patterned area, and were able to learn reward location (Fig. 2c, d). This confirms that a contrasting visual floral guide, as in the dark blue patterns, could induce all the responses associated with guides in bees visiting the experimental artificial flowers. This suggests that the lack of all these guide-associated responses by bees visiting flowers with temperature patterns is not because the bees are unable to show them in our experiment, but due to the reduced efficiency of temperature pattern cues as floral guides. These results suggest that temperature patterns can convey messages of within-flower reward location to bumblebees, functioning like floral guides. However, temperature patterns appear to function differently, inducing changes in bumblebee flower handling but not eliciting all the responses which contrasting colour patterns would. This differing functionality of temperature and visual guides highlights the importance of considering many aspects of flower handling (here the handling metrics) when considering the capacity of floral patterns to function as guides.

The addition of temperature patterns to artificial flowers reduced incidence of failed visits and improved floral reward location learning. These changes in bumblebee behaviour occurred when temperature patterns were presented alone (Unimodal groups), and when temperature patterns were combined with colour patterns (Multimodal groups) in addition to any behaviours elicited by the colour patterns alone. The addition of a warm temperature pattern to a blue pattern in the Multimodal Blue flowers resulted in further reductions in proportions of failed visits, compared to the Unimodal Blue group bees (Fig. 2a, b), and further enhanced learning of reward location, as shown by higher proportions of first-feeder visits achieved (Fig. 2c, d). However, the addition of temperature patterns to a blue colour guide did not enhance initial proportion of first-feeder visits of bees visiting multimodal flowers (Fig. 2c, d). Similarly, reward search time was not enhanced by the addition of temperature to a blue colour pattern (Fig. 2e, f). In these latter cases, bees responded similarly to bees presented with blue colour patterns alone (and this similarity between unimodal and multimodal flowers was true for all measures of enhancement in pink flowers). These results show overlapping thermal and visual guides function similarly to the sum of their unimodal components, in terms of the handling responses elicited in bumblebees (the various forms of ${y}_{nx}$, equation S5 in Appendix S3). We can conclude from our results that temperature patterns, when added to a dark contrasting visual pattern, may enhance flower handling as a multimodal guide.

Bees showed consistent improvements with experience in reward search time (Fig. 2e, f) and proportions of failed visits (Fig. 2a, b) even in non-patterned control groups, or in flowers with apparently undetectable Pink patterns. However, these responses were improved further by the presence of certain patterns, and bees required pattern signals to show changes in proportions of first-feeder visits. The decrease in proportions of failed visits with increased experience independent of pattern signal presence is probably the result of bees learning that rewards are always present in the experimental flowers. Even though bees in control groups cannot distinguish which feeder is rewarding, their motivation to stay increases with experience (Lefebvre et al. 2007; Taneyhill 2010). They therefore become more likely to continue to search after a nonrewarding feeding attempt. The increase in reward search time independent of pattern signal is the result of bees learning how to better access feeders in tunnels. This leads to faster reward search time, as bees in the control group more quickly investigate each tunnel.

Based on bumblebees’ preferences for temperature (Dyer et al. 2006; Whitney et al. 2008; Norgate et al. 2010), we predicted that bees would be innately attracted to temperature patterns corresponding with the rewarding feeder and would therefore visit that feeder first, leading to a higher initial proportions of first-feeder visits and reduced reward search times. However, bees appeared to have to learn the temperature pattern guide to show improved proportions of first-feeder visits using temperature patterns and no temperature related benefit to reward search time was seen. Proportions of failed visits were consistently reduced in flowers with Warm temperature patterns, suggesting that naïve bees are more likely to find rewards but are not more likely to correctly choose to approach the correct location first. The decreased proportions of failed visits, but unaffected initial proportions of first-feeder visits or reward search time of naïve bees in response to temperature patterns, is probably the result of spatial range of floral temperature signals. In bumblebees, temperature is detected by conduction or touch (Dyer et al. 2006; Whitney et al. 2008). Unlike with visual signals (Lunau et al. 2006), bees are not informed or attracted to the reward location until they have landed and actually made contact with the temperature pattern during their search. Naïve bees are likely to land and search similarly to control group bees until they actually encounter the temperature patterns, and are therefore more likely to erroneously approach and feed from nonrewarding feeders. When presented with Warm temperature patterns, the lower incidence of failed visits of naïve bees indicates that they are more likely to feed from the warm feeder once they encounter the pattern (Angioy et al. 2004; Dyer et al. 2006). However, as bees do not detect the temperature pattern until they have searched the flower we saw no improvement in incidence of first-feeder visits in naïve bees or in naïve bee reward search times. The lack of improvement in bee incidence of failed visits when presented with Hot temperature patterns may be due to the excess heat discouraging bees. Although bees prefer higher floral temperature, there is a point where flowers will become too hot and will deter bees (as demonstrated in Australian stingless bees Norgate et al. 2010; Shrestha et al. 2018). It is possible the Hot temperature patterns we used were sufficiently hot to deter naïve bumblebees, meaning that they would have been less likely to search the rewarding feeder when they encountered it—further experimentation is required to explore this suggestion.

Multimodality has been observed to enhance learning of differences between flowers (Kulahci et al. 2008; Katzenberger et al. 2013), perhaps further when different modality patterns overlap (Lawson et al. 2018). Our results suggest multimodality may also enhance within flower learning; this would explain the further improvements in first feeder visits in bees visiting Multimodal Blue flowers compared to Unimodal Blue group bees. Similarly, the attractive properties of temperature patterns and colour patterns combined further improve the chances of bees feeding on the rewarding feeder when they encounter it with the multimodal pattern, resulting in the greater reduction in failed visits. Reward search time and the initial first-feeder visit probabilities are not further enhanced in multimodal group bees. This is likely to be a result of temperature pattern’s reduced spatial range, meaning that naïve Multimodal Blue group bees land and search flowers similarly to Unimodal Blue bees until they encounter temperature patterns. Combinations of other floral pattern modalities have not previously been observed to enhance floral guide functionality. Lawson et al. (2017a) found multimodal scent and colour patterns did not enhance bumblebee reward search times any more than the pattern’s unimodal components would alone. Similarly, Goyret (Goyret 2010) and Goyret and Kelber (2011) found that nocturnal and diurnal moths respond to multimodal tactile and colour guides in the same manner as they do to unimodal tactile and colour guides respectively. The results presented here support the findings of Lawson et al. (2017a), where bee reward search times were not enhanced in multimodal guides (Fig. 2e, f). However, our findings show that multimodality enhances guide function in terms of other handling metrics. Perhaps multimodality may have additive effects in some aspects of flower handling, but not reward search time. This highlights the importance of considering multiple aspects of within flower foraging behaviour floral guides might influence, particularly when considering pollinator responses to multimodal patterns. Alternatively, the different effectiveness of multimodal guides seen here may reflect differences in how bees respond to combinations of different signalling modalities. Lawson et al. (2017a) found no additional effects of multimodality on reward search time even though both unimodal visual and scent guides alone influenced reward search time. Recent research has found scent and temperature patterns to interact differently with visual patterns (Lawson et al. 2018; Harrap et al. 2019), hinting that spatial information from visual and scent patterns may be processed together in the bumblebee’s brain, while temperature patterns appear to be processed separately from visual patterns. Such differing interactions between modalities may explain the differing effectiveness of different multimodal guides. Furthermore, these differing effects of combining patterns suggests that temperature patterns work differently as floral guides to visual patterns (and perhaps to scent patterns as well).

Temperature patterns are common across the flowering plants especially alongside darker pigmentation, such as those seen in many visual guides (Harrap et al. 2017). The responses of bees to artificial temperature patterns would suggest that in natural flowers temperature patterns, either as unimodal patterns or alongside visual patterns, might ensure that more visitors reach the correct position for pollen transfer, enhancing plant fitness. Additionally, the reductions in failed visits and improvement in learning the location of rewards would improve bee foraging efficiency. This will have consequential effects on bee flower choice, and thus also plant fitness. This would be particularly true on complex flowers where learning the correct position to find rewards is a harder task for the bee. Given that differing temperature patterns across the surface of the flower are common in flowering plants, bees will have the opportunity to experience these patterns, and are therefore unlikely to remain naïve to temperature patterns while foraging in natural environments. As seen in colour guides, learning and guided responses can carry over to novel displays with similar guides (Leonard and Papaj 2011). This may also occur with temperature patterns. Understanding how previous experience on displays with similar patterns influences bee responses on novel flowers may be important to understanding how temperature patterns, and other floral guides, influence bee behaviour within natural flowers.

As darker pigmented colour guides will often warm up in sunlight (Harrap et al. 2017) the foraging efficiency and pollen transfer benefits of visual guides may be further reinforced by these co-occuring floral temperature patterns. However, other explanations of multimodality beyond the ‘multiple messages hypothesis’ exist (Leonard et al. 2012) that may result in further fitness benefits from possession of both thermal and visual patterns together. These should also be considered. Learning of reward location was enhanced by possession of both temperature and visual patterns (Fig. 2c, d), and learning of flower identify may also be improved. Bumblebees can learn both thermal and visual patterns and use these for flower identification, and so flower recognition may also be improved by possessing both (Kulahci et al. 2008; Kaczorowski et al. 2012; Leonard and Masek 2014). Having both guide signals may be of further benefit if one is disrupted by environmental conditions mitigating the effects of signal disruption (the efficacy backup hypothesis—Leonard et al. 2012; Kaczorowski et al. 2012; Lawson et al. 2017b). The responses to Multimodal Pink flowers suggest that temperature patterns provide benefits to flower handling when visual patterns are non-functioning. However, both colour pattern recognition by pollinators (Dyer and Chittka 2004a, b) and floral temperature pattern generation (Rejšková et al. 2010) are influenced by light conditions, so it is likely both patterns will be disrupted together rather than independently (although temperature could be disrupted differently in environments where dew or precipitation occur: Lawson and Rands 2019). Bumblebees have approximate colour constancy, meaning that they confuse similar colours in altered light conditions (Chittka et al. 2014), but may still remain sensitive to small differences in temperature (Heran 1952; Dyer et al. 2006). The extent of temperature pattern generation disruption alongside changes of light conditions needs further investigation to assess the effects of meaningful environmental changes, and evaluate the potential benefit of thermal and colour patterns on efficacy backup.

Bee responses to temperature patterns within the flower reveal that temperature patterns can signal reward location, therefore, functioning like a floral guide. Floral temperature patterns appear to perform this role differently to contrasting colour guides. This expands the potential functionality of floral temperature and patterns of temperature within a floral display. The current study also suggests that temperature patters can provide benefits to flower handling alongside contrasting colour patterns, suggesting temperature and visual patterns that occur together function as a multimodal guide. However, we support previous evidence that bee reward search times are not influenced by multimodal patterns beyond responses shown to unimodal components. Based on the findings of this study and existing work, it would appear that the effective range of the floral guide signal has an important effect on both how well different modalities function as guides as well as how bees utilise different guides within a multimodal display.

## Electronic supplementary material

Below is the link to the electronic supplementary material.

Supplementary file1 (DOCX 15 kb)

Supplementary file2 (DOCX 3497 kb)

Supplementary file3 (CSV 3 kb)

Supplementary file4 (CSV 13 kb)

Supplementary file5 (CSV 165 kb)
