# Supplementary material for: Floral temperature patterns can function as floral guides
Source: Arthropod Plant Interact. 2020 Jan 13;14(2):193–206. doi: 10.1007/s11829-020-09742-z (PMC7073333; doi:10.1007/s11829-020-09742-z)
Supplement: Supplementary file 1 — Supplementary file1 (DOCX 15 kb) [file 11829_2020_9742_MOESM1_ESM.docx]

**Floral temperature patterns can function as floral guides**

**Key to datasheets**

**Michael J. M. Harrap***^1^, Natalie Hempel de Ibarra^2^, Heather M. Whitney^1^, Sean A. Rands^1^

MJH: m.j.m.harrap@gmail.com <http://orcid.org/0000-0003-0515-2348>

NHI: n.hempel@exeter.ac.uk <http://orcid.org/0000-0002-0859-8217>

HMW: heather.whitney@bristol.ac.uk <http://orcid.org/0000-0001-6450-8266>

SAR: sean.rands@bristol.ac.uk <http://orcid.org/0000-0002-7400-005X>

***author for correspondence**

Three datasheets are attached.

**Visit data**

The observations taken at each focal flower visit made by each bee, extracted in video processing.

Column designations are as follows:

#: an individual visit counter, for data management

Bee: bee identifier number within the experiment. Runs from 1 to 97. Due to a counting error there is no bee 44, thus 96 bees in total.

nest: The nest the bee was from. Nests are identified by the lettering indicating the workstation within the Lab their flight area was on. If a nest is replaced a number is added to the workstation letter (*e.g.* E2).

Test: The test group the bee was in. Test groups are described in the main text. Labelling is as follows: ‘Acontrol’= Plain Control test group; ‘Plate Acontol’= Plates Control test group; ‘HOT’= Unimodal Hot test group; ‘Pink Plates’= Unimodal Pink test group; ‘Blue Plates’ = Unimodal Blue test group; ‘Warms’= Unimodal Warm test group; ‘MultimodalPink’= Multimodal Pink test group; ‘MultiModalBlue’= Multimodal Blue test group.

Date: the date of the bee’s test phase trial, stylized as *DD/MM/YYYY*.

Flower Visit: The focal flower visit of that bee.

cam# - video: The video camera and video ID number on which the focal visit was recorded.

Rewards found: An indicator as to whether bees found rewards during that focal visit or not. If ‘y’ bees found rewards in this focal visit. If ‘n’ bees failed to find rewards.

# of feeders used (0 to 3): the number of feeders the bee drank from in that focal visit. Can be values of either ‘1’, ‘2’ or ‘3’ (3 being to total number of feeders on the flower), or ‘n/a’ if bees did not drink from any feeder.

rewards were the # feeder used (0 to 3): how many feeders bees needed to attempt to drink from before finding the rewarding feeder on this focal visit. Values can be: ‘n/a’ if bees failed to find rewards; ‘1’ bees found rewards on the first feeder they attempted to drink from; ‘2’ bees found rewards on the second feeder they attempted to drink from; ‘3’ bees found rewards on the third feeder they attempted to drink from.

Time until bee found rewards (sec): The reward search time until bees found rewards on this focal visit.

**Probability data**

The probability data calculated for each bee as described in the main text using the observations made at each focal visit.

Column designations are as follows:

Bee, nest, Test and date: as described above.

last: The focal visit number at which probabilities were calculated as described in the main text.

visits in division (division counter): The number of focal visits used to calculate probabilities, this is normally 10 visits but, as described in the main text, on certain bees 9 visits are used.

# failed to find rewards: the number of visits in the previous 10 visits where the bee failed to find rewards.

proportion of failed to find rewards: The proportion of failed visits in the previous 10 visits made by each bee. As described in the main text.

# first use being sucrose: the number of visits in the previous 10 visits where the rewarding feeder was the first bees attempted to drink from

proportion of failed to find rewards: The proportion of first-feeder visits in the previous 10 visits made by each bee. As described in the main text.

**Plotted points**

Provides detail on the points plotted in respective panels within fig 2. For fig 2a, 2b, 2c and 2d the means and SEM values, as described in the figure legend are given. For fig 2e and 2f the formula for the mean line and SEM is given for each test group or patterns, as described in the figure legend.
