# Supplementary material for: Floral temperature patterns can function as floral guides
Source: Arthropod Plant Interact. 2020 Jan 13;14(2):193–206. doi: 10.1007/s11829-020-09742-z (PMC7073333; doi:10.1007/s11829-020-09742-z)
Supplement: Supplementary file 2 — Supplementary file2 (DOCX 3497 kb) [file 11829_2020_9742_MOESM2_ESM.docx]

**Floral temperature patterns can function as floral guides**

**Supplementary Material**

**Michael J. M. Harrap***^1^, Natalie Hempel de Ibarra^2^, Heather M. Whitney^1^, Sean A. Rands^1^

MJH: m.j.m.harrap@gmail.com <http://orcid.org/0000-0003-0515-2348>

NHI: n.hempel@exeter.ac.uk <http://orcid.org/0000-0002-0859-8217>

HMW: heather.whitney@bristol.ac.uk <http://orcid.org/0000-0001-6450-8266>

SAR: sean.rands@bristol.ac.uk <http://orcid.org/0000-0002-7400-005X>

***author for correspondence**

**Appendix S1. The reflectance spectra of the artificial flowers**

The reflectance spectra of the aqua blue plastic and Control, Blue and Pink panels stuck onto aqua blue plastic are presented in figure S1. The excitation that these colours would induce on bee colour receptors was calculated for average daylight conditions. The difference between colours to the bee could then be expressed as distance from the aqua blue background in bees’ 2D colour hexagon space, their ‘hexagon units’ as described in Dyer and Chittka (2004a). Control panels showed a distance of 0.01 hexagon units (to 2 d.p.) from background; Pink panels a distance of 0.02 hexagon units from the background, and Blue panels a distance of 0.04 hexagon units. This suggests that to the bees, blue panels show twice as much contrast with the background as Pink panels, Control panels showing even less contrast.

**Fig. S1** The reflectance spectra of the PVC plastics used in artificial flower construction. These are measured using an Ocean Optics, Flame-S-UV-VIS miniature spectrometer (Ocean optics, Largo, USA), with a deuterium-halogen tungsten lamp (DH-2000-BAL UV-VIS-NIR, Ocean optics, Largo, USA) as light source and a QR400-7-UV-VIS reflection probe (Ocean optics, Largo, USA). All spectra measurements were taken against a white Spectralon standard (WS-1-SL Ocean optics, Largo, USA). All panels are measured stuck to a sheet of the background plastic. Reflectance is given as a ratio of the Spectralon standard. Colour, labels, and dashing of spectra indicate the plastic colour. Aqua blue lines, labelled AB, indicate the spectra of the aqua blue plastic. The background sheet when dotted, and the Control panel plates when solid. Dark blue dashed line, labelled DB, is the spectra of the dark blue plates. Pink, dash-dot line, labelled P, is the spectra of the pink plates


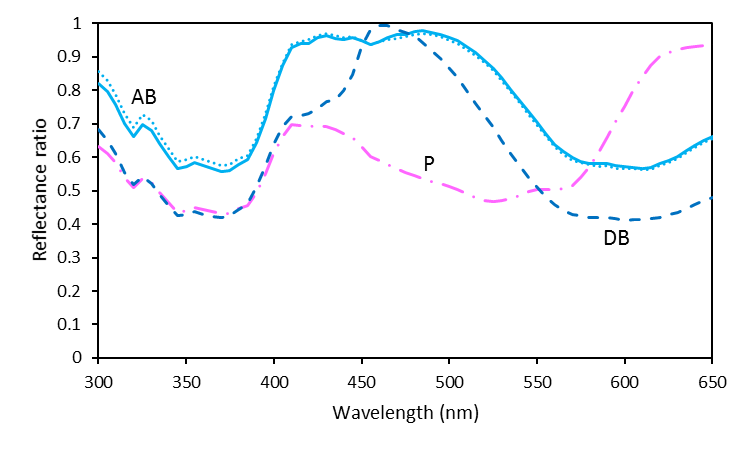


**Appendix S2. Construction of heated pattern elements of artificial flowers**

Heating elements for the ‘Hot’ temperature pattern were created by cutting a 2×3cm section of 5mm neoprene foam (Camthorne Industrial Supplies Ltd, Staffordshire, UK) and a further two sheets of 1mm plastic foam were glued to underside of the flower top, just in front of one feeding well. These sheets were stuck so that it faced outwards from the centre of the flower towards a feeder (Fig. S2a). A 13cm length of 0.32mm, 17.87Ωm^-1^ kanthal resistance wire was bent into an M shape with two 1cm leads on each end. This wire was covered and stuck down by a pressure sensitive putty (*Blu Tack*: Bostik, Paris, France), and the wire leads attached to an AA battery cradle using sections of a connector block (Fig. S2b). When a battery was inserted into the cradle, the area above and around the heat sink heated up. This warmed a third of the artificial flower, and was hottest in front of the tunnel above the heating elements (Fig. 1e).

‘Warm’ temperature patterns were made in a similar way as ‘Hot’ patterns. Here the whole underside of the flower top was insulated with a 76mm diameter disc of 5mm neoprene foam. Four 3×2cm sheets of 1mm neoprene foam were stuck on top of each other to the underside of this disc, and placed so that it led from the flower centre towards a tunnel (Fig. S2c). A 20cm section of resistance wire was bent into an alternating zig-zag shape to fit on the 3×2cm space, allowing 1cm leads on each end, and was then covered and stuck down with Blu Tack and connected a battery cradle as described above (Fig. S2d). The ‘Warm’ temperature pattern heating elements created a bar of warmer temperature localised in the area in front of the feeder tunnel (Fig. 1f).

**Fig. S2.** Details of heated flower construction. **a** An underside view of the heat sinks used to generate a Hot temperature guide. **b** Hot guide flowers undersides with heating elements attached. **c** An underside view of the heat sinks used to generate a Warm temperature guide. **d** Warm guide flowers undersides with heating elements attached.


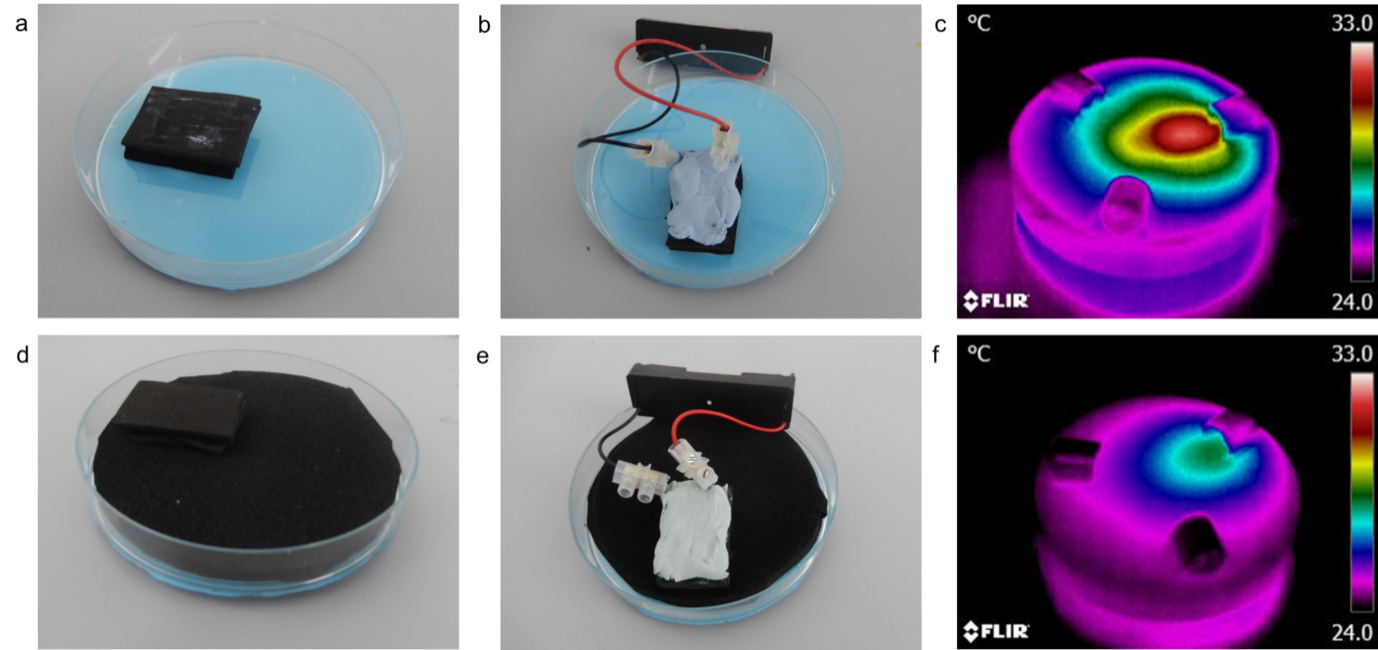


c

d

a

b

**Appendix S3. Detail of statistical analyses**

Before the effect of patterns were analysed, bee responses in the Panels Control and Plain Control flower were compared. This allowed us to assess whether these groups could be treated as equivalent, greatly simplifying later models. The full model used to compare Control test groups responses (before any simplification applied) was

$y_{nx}=i_{a}+(v*l_{a})+G\left( i_{b}+\left( v*l_{b} \right) \right)+b_{n}+\left( v*r_{n} \right)$. (S1)

Here $y_{nx}$ is the bee handling metric (the arcsine transformed failure rate, arcsine transformed first-feeder rate or reward search time in seconds) of bee $n$ on focal visit number $x$, where $x$ is the focal visit number in bee $n$’s sequence of visits. $v$ relates to focal visit $x$ by the following. For reward search time

$v=\ln x$*,* (S2)

for failure and first feeder rates

$v=ln(x+1-10)$. (S3)

The transformations shown in equations S2 and S3 allow the model to show a logarithmic relationship. $i_{a}$ is the intercept, the initial value of the metric in question, for bees in the Plain Control group. $l_{a}$ represents the change in $y_{nx}$ of Plain Control bees with experience, increased values of $v$ and thus also $x$, indicating bee learning speed. Parameter $i_{b}$ represents the change in model intercept, relative to $i_{a}$, for bees in the Panels Control group. Parameter $l_{b}$ represents the change in learning speed, relative to $l_{a}$, for bees in the Panels Control group. These modifications are applied to bees in test group $b$ by operator $G$,

$G=\left\{ \begin{matrix} 0, \\ 1, \end{matrix} \begin{matrix} bee is in Plain Control group \\ bee is in Panels Control group \end{matrix} \right.$ . (S4)

Random factors are included in this model with $b_{n}$ and $r_{n}$, that represent the change in model intercept and learning speed of bee $n$, relative to $i_{a}$ and $l_{a}$. When comparing Control groups, the initial standing best model was as given in equation S1. This was compared with a model where $l_{b}$ was removed, removing different learning speeds between the Control groups; interaction between experience and test group. If this had no effect on AIC the new standing best model (without $l_{b}$) was compared to one where differences in intercepts were removed, by removing $i_{b}$. A significant effect of either parameter implied that the two Control groups differ.

A different model was used to compare bee responses to patterns. This model allowed common effects of patterns, particularly those that make up multimodal patterns, to be assessed across test groups. This accounts for a common response to the same pattern across different test groups. The full model applied to failure rate, first-feeder rate and reward search time before any model simplification is as follows

$y_{nx}=i+\left( v\cdot l \right)+b_{n}+\left( v\cdot r_{n} \right)+P\left( i_{p}+\left( v\cdot l_{p} \right) \right)+B\left( i_{b}+\left( v\cdot l_{b} \right) \right)$

$$+W\left( i_{w}+\left( v\cdot l_{w} \right) \right)+H\left( i_{h}+\left( v\cdot l_{h} \right) \right)+P\cdot W\left( i_{pw}+\left( v\cdot l_{pw} \right) \right)$$

$$+B\cdot W\left( i_{bw}+\left( v\cdot l_{bw} \right) \right) .$$

(S5)

Equation S5 does not include any effects of panels on bee responses (as none were detected in the above control test group comparisons). Here parameters $y_{nx}$ and $v$ work as described above. $i$is the model intercept, the initial value of $y_{nx}$ when $v=0$, for bees presented with no patterns. $l$ represents the change in $y_{nx}$ relative to$i$ with increased values of $v$ for bees presented with no patterns, and therefore represents the learning speed. Random factors $b_{n}$ and $r_{n}$ work as above but now refer to changes in model intercept and learning speed in bee $n$ relative to $i$ and$l$. $i$ and $l$ are modified when bees are presented with flowers which have floral patterns. This is controlled through operators $P$,$B$, $W$ and $H$, where

$P=\left\{ \begin{matrix} 0, \\ 1, \end{matrix} \begin{matrix} flower presented to bee n does not have a Pink colour pattern \\ flower presented to bee n does have a Pink colour pattern \end{matrix} \right.$ (S6)

$B=\left\{ \begin{matrix} 0, \\ 1, \end{matrix} \begin{matrix} flower presented to bee n does not have a Blue colour pattern \\ flower presented to bee n does have a Blue colour pattern \end{matrix} \right.$ (S7)

$W=\left\{ \begin{matrix} 0, \\ 1, \end{matrix} \begin{matrix} flower presented to bee n does not have a Warm thermal pattern \\ flower presented to bee n does have a Warm thermal pattern \end{matrix} \right.$ (S8)

$H=\left\{ \begin{matrix} 0, \\ 1, \end{matrix} \begin{matrix} flower presented to bee n does not have a Hot thermal pattern \\ flower presented to bee n does have a Hot thermal pattern \end{matrix} \right.$ . (S9)

Pattern-induced changes in intercept and learning speed relative to$i$ and $l$ are given by: $i_{p}$ and $l_{p}$ when bees are presented with Pink colour patterns; $i_{b}$ and $l_{b}$ when bees are presented with Blue colour patterns; $i_{w}$ and $l_{w}$ when bees are presented with Warm temperature patterns; and $i_{h}$ and $l_{h}$ when bees are presented with Hot temperature patterns. When bees are presented with a combination of patterns as in the multimodal groups the model will apply both patterns’ induced changes (see equation S5) but will also apply further specific multimodal changes. The parameters $i_{pw}$ and $l_{pw}$ represent the change in model intercept and learning relative to $i$ and$l$ for bees presented with Warm and Pink patterns (*i.e*. those in the Multimodal Pink group). Likewise, parameters $i_{bw}$ and $l_{bw}$ represent the change in model intercept and learning relative to $i$ and $l$ for bees presented with Warm and Blue patterns (i.e. those in the Multimodal Blue group). With these multimodal interaction effects, the model may magnify bee responses to multimodal flowers if positive, alternatively these parameters can reduce or remove the additive benefits of additional patterns if negative. If these values equal zero the model allows the effects of one pattern to be added to the other with no further modification. Note that due to the arcsine transformations applied to proportions these effects will not be truly additive, as they will be reduced as bees approach values of 1 or 0.

The model simplification process applied to the model in equation S5 is as follows. The initial standing best model was as given in equation S5. This was compared to a model where individual bee learning speeds ($r_{n}$) were removed. The standing best model was then compared to one where parameter $l$ was removed. Testing the effects of $l$ first was necessary as it established what responses control group bees gave. This ensured subsequent comparisons were made against bees in the control group, or groups responding the same way as in the control group. Next, the effect of removal of multimodal interaction effects of bees presented with the Pink Multimodal flowers, removing parameters $i_{pw}$ and $l_{pw}$. Then the same effects on bees presented with Blue multimodal flowers, removing parameters $i_{bw}$ and $l_{bw}$ was tested. After this each pattern’s individual effects was investigated. Whether each pattern alters the learning speed of bees was tested by comparing the standing best model to one where that patterns’ modifier of $l$ ($l_{p}$, $l_{b}$, $l_{w}$, and$l_{h}$ respectively) was removed. The standing model after that comparison was used to investigate whether each pattern alters the initial response, intercept, of bees by comparing the best model to one where that patterns’ modifier of $i$ ($i_{p}$, $i_{b}$, $i_{w}$, and $i_{h}$ respectively) was removed. These two comparisons were carried out for each pattern in the following order: Pink, Blue, Warm and Hot. Model intercept $i$ and random individual effect on bee intercepts ($b_{n}$) were not assessed and remained in the model.

**Table S1** The results of model selection for bee proportions of failed visits. Comparisons of standing best models and a simpler version where a focal parameter is removed are given for each effect tested in our model selection process. AIC is given for both models (note how standing best model AIC matches one of the previous model AICs. Also given is an assessment of model fit, Δdeviance=Δdev. A verdict on each comparison is given: Asterisks ‘✱’ indicate parameters where models including them have lower AIC, based on Richards (2008), and are thus included in best model. Note that the AIC criteria of Richards (2008) require a ΔAIC of at least 6 for a more complex models to be favoured. More complex models may still have better fit, but be comparable in terms of AIC. Our model inclusion was based on AIC. The p values of parameters where inclusion significantly improved fit but not AIC sufficiently are labelled with a ‘^†^’.

| Tested effect |  | Standing best model AIC | Simpler model AIC | ΔAIC | Δdev. | df | *p* | Verdict | Value |
| --- | --- | --- | --- | --- | --- | --- | --- | --- | --- |
| Model Intercept | $i$ |  |  |  |  |  |  |  | 0.70 |
| Individual learning speeds | $r_{n}$ | -15.88 | -19.27 | 3.39 | 0.61 | 2 | 0.737 |  |  |
| Background learning in Controls | $l$ | -19.27 | -3.88 | 15.40 | 17.40 | 1 | <0.01 | ✱ | -0.09 |
| Pink Multimodal Interaction Effects | $i_{pw}$ $l_{pw}$ | -19.27 | -22.56 | 3.29 | 0.71 | 2 | 0.701 |  |  |
| Blue Multimodal Interaction Effects | $i_{bw}$ $l_{bw}$ | -22.56 | -22.99 | 0.42 | 3.57 | 2 | 0.167 |  |  |
| Altered learning of Pink Patterns | $l_{p}$ | -22.99 | -24.52 | 1.53 | 0.47 | 1 | 0.493 |  |  |
| Altered intercepts of Pink Patterns | $i_{p}$ | -24.52 | -25.07 | 0.55 | 1.45 | 1 | 0.229 |  |  |
| Altered learning of Blue Patterns | $l_{b}$ | -25.07 | -25.04 | 0.03 | 2.03 | 1 | 0.154 |  |  |
| Altered intercepts of Blue Patterns | $i_{b}$ | -25.04 | 0.53 | 25.57 | 27.57 | 1 | <0.01 | ✱ | -0.19 |
| Altered learning of Warm Patterns | $l_{w}$ | -25.04 | -26.98 | 1.94 | 0.06 | 1 | 0.811 |  |  |
| Altered intercepts of Warm Patterns | $i_{w}$ | -26.98 | -5.29 | 21.69 | 23.70 | 1 | <0.01 | ✱ | -0.15 |
| Altered learning of Hot Patterns | $l_{h}$ | -26.98 | -27.66 | 0.68 | 1.32 | 1 | 0.251 |  |  |
| Altered intercepts of Hot Patterns | $i_{h}$ | -27.66 | -23.25 | 4.42 | 6.42 | 1 | 0.011^†^ |  |  |

**Table S2:** The results of model selection for proportions of first-feeder visits. Comparisons of standing best models and a simpler version were parameters are removed are given in for each effect tested in our model selection process. AIC is given for both models (note how standing best model AIC matches one of the previous model AICs. Also given is an assessment of model fit, Δdeviance=Δdev. A verdict on each comparison is given: Asterisks ‘✱’ indicate parameters where models including them have lower AIC, based on Richards (2008), and are thus included in best model. For fixed effect parameters that are in best model their value in the best model is given, with reference to equation 5.

| Tested effect |  | Standing best model AIC | Simpler model AIC | ΔAIC | Δdev. | df | *p* | Verdict | Value |
| --- | --- | --- | --- | --- | --- | --- | --- | --- | --- |
| Model Intercept | $i$ |  |  |  |  |  |  |  | 0.67 |
| Individual learning speeds | $r_{n}$ | -86.15 | -76.64 | 9.51 | 13.51 | 2 | <0.01 | ✱ | - |
| Background learning in Controls | $l$ | -86.15 | -88.10 | 1.95 | 0.05 | 1 | 0.830 |  |  |
| Pink Multimodal Interaction Effects | $i_{pw}$ $l_{pw}$ | -88.10 | -91.02 | 2.91 | 1.09 | 2 | 0.581 |  |  |
| Blue Multimodal Interaction Effects | $i_{bw}$ $l_{bw}$ | -91.02 | -93.33 | 2.31 | 1.69 | 2 | 0.429 |  |  |
| Altered learning of Pink Patterns | $l_{p}$ | -93.33 | -94.55 | 1.23 | 0.77 | 1 | 0.379 |  |  |
| Altered intercepts of Pink Patterns | $i_{p}$ | -94.55 | -96.32 | 1.76 | 0.24 | 1 | 0.626 |  |  |
| Altered learning of Blue Patterns | $l_{b}$ | -96.32 | -69.72 | 26.59 | 28.59 | 1 | <0.01 | ✱ | 0.10 |
| Altered intercepts of Blue Patterns | $i_{b}$ | -96.32 | -88.59 | 7.72 | 9.72 | 1 | <0.01 | ✱ | 0.14 |
| Altered learning of Warm Patterns | $l_{w}$ | -96.32 | -87.35 | 8.97 | 10.97 | 1 | <0.01 | ✱ | 0.07 |
| Altered intercepts of Warm Patterns | $i_{w}$ | -96.32 | -97.01 | 0.70 | 1.31 | 1 | 0.253 |  |  |
| Altered learning of Hot Patterns | $l_{h}$ | -97.01 | -90.07 | 6.94 | 8.94 | 1 | <0.01 | ✱ | 0.06 |
| Altered intercepts of Hot Patterns | $i_{h}$ | -97.01 | -97.75 | 0.74 | 1.26 | 1 | 0.261 |  |  |

**Table S3:** The results of model selection for bee reward search times rates. Comparisons of standing best models and a simpler version were parameters are removed are given in for each effect tested in our model selection process. AIC is given for both models (note how standing best model AIC matches one of the previous model AICs. Also given is an assessment of model fit, Δdeviance=Δdev. A verdict on each comparison is given: Asterisks ‘✱’ indicate parameters where models including them have lower AIC, based on Richards (2008), and are thus included in best model. Note that the AIC criteria of Richards (2008) require a ΔAIC of at least 6 for a more complex models to be favoured. More complex models may still have better fit, but be comparable in terms of AIC. Our model inclusion was based on AIC. The p values of parameters where inclusion significantly improved fit but not AIC sufficiently are labelled with a ‘^†^’.

| Tested effect |  | Standing best model AIC | Simpler model AIC | ΔAIC | Δdev. | df | *p* | Verdict | Value |
| --- | --- | --- | --- | --- | --- | --- | --- | --- | --- |
| Model Intercept | $i$ |  |  |  |  |  |  |  | 9.97 |
| Individual learning speeds | $r_{n}$ | 14241 | 14291 | 50 | 54.06 | 2 | <0.01 | ✱ | - |
| Background learning in Controls | $l$ | 14241 | 14256 | 15 | 16.68 | 1 | <0.01 | ✱ | -1.71 |
| Pink Multimodal Interaction Effects | $i_{pw}$ $l_{pw}$ | 14241 | 14237 | 4 | 0.22 | 2 | 0.897 |  |  |
| Blue Multimodal Interaction Effects | $i_{bw}$ $l_{bw}$ | 14237 | 14235 | 2 | 2.12 | 2 | 0.346 |  |  |
| Altered learning of Pink Patterns | $l_{p}$ | 14235 | 14234 | 1 | 1.06 | 1 | 0.302 |  |  |
| Altered intercepts of Pink Patterns | $i_{p}$ | 14234 | 14237 | 3 | 4.55 | 1 | 0.033^†^ |  |  |
| Altered learning of Blue Patterns | $l_{b}$ | 14237 | 14238 | 1 | 3.26 | 1 | 0.071 |  |  |
| Altered intercepts of Blue Patterns | $i_{b}$ | 14238 | 14278 | 40 | 41.54 | 1 | <0.01 | ✱ | -2.28 |
| Altered learning of Warm Patterns | $l_{w}$ | 14238 | 14236 | 2 | 0.00 | 1 | 0.961 |  |  |
| Altered intercepts of Warm Patterns | $i_{w}$ | 14236 | 14235 | 1 | 1.12 | 1 | 0.289 |  |  |
| Altered learning of Hot Patterns | $l_{h}$ | 14235 | 14234 | 1 | 0.34 | 1 | 0.560 |  |  |
| Altered intercepts of Hot Patterns | $i_{h}$ | 14234 | 14233 | 1 | 1.04 | 1 | 0.307 |  |  |
